# Optimization of Filling Material Ratio in Yellow Phosphorus Slag Mine

**DOI:** 10.3390/ma17225521

**Published:** 2024-11-12

**Authors:** Tao Deng, Bokai Xia, Yuanhuan Liao, Chengliang Zhang

**Affiliations:** Faculty of Land Resources Engineering, Kunming University of Science and Technology, Kunming 650500, China; 20120165@kust.edu.cn (T.D.); 13629068228@163.com (B.X.); 13092810873@163.com (Y.L.)

**Keywords:** yellow phosphorus slag, filling strength, packing density models, mixed filling material, filling value calculation

## Abstract

Yellow phosphorus slag has been considered as a potential cement substitute for mine filling material due to its cementing activity; however, its slow setting and low early strength have limited broader use. This study investigates the grading, compactness, and strength of yellow phosphorus slag combined with tailing sand. Using yellow phosphorus slag as an aggregate, cement as a binder, and mixing tailing sand in different ratios, this study evaluates its feasibility as a coarse aggregate in mine backfill. The key findings are as follows. (1) The grading index of tailing sand was 0.5, aligning with Fuller grading, but it required mixing with coarse aggregates to enhance strength and reduce cement consumption. Yellow phosphorus slag, with a grading index of 0.97, does not match Fuller’s curve and thus benefits from mixing with tailing sand. (2) For mixtures of waste rock and tailings, the 5:5 ratio aligned closely with Fuller’s theory, showing optimal packing density and strength. Mixtures of yellow phosphorus slag and tailings at ratios of 3:7, 4:6, and 5:5 had R^2^ values of 0.73, 0.80, and 0.85, respectively, confirming reliable fit. The 5:5 mixture provided the best packing density and strength. (3) A new strength prediction model, accounting for aggregate, cement, and water effects, suggests that a 5:5 ratio with a 71% mass concentration and 1/7 ash–sand ratio meets industrial strength requirements. FLAC3D simulations indicated that cemented backfill reduces stress concentrations caused by excavation and supports stability during mining while also absorbing energy through compaction, creating favorable conditions for safe mining operations.

## 1. Introduction

As a key technology within the concept of ‘green mining’, backfill mining offers several advantages, including reduced mining and excavation, high flexibility, and minimal ore loss and dilution rates [1,2,3]. This method can eliminate surface subsidence caused by mining activities, improve the mining stress environment, and reduce the discharge of solid wastes such as waste rock and tailings [4,5,6]. Mineral deposits that can accommodate various mining technologies, especially those associated with ‘three-down mining’, ‘large water’, and ‘complex’ mineral deposits, align with the current scientific concept of sustainable development. These deposits essentially fulfill the three criteria of high comprehensive resource utilization efficiency, minimal waste generation, and surface preservation [7,8,9,10]. Undoubtedly, the filling mining method stands as the top choice in the context of green mining.

### The Application and Research Status of Yellow Phosphorus Slag in Filling Mining

Yellow phosphorus slag is produced through the electric furnace method of extracting yellow phosphorus from phosphorus ore, silica, coke, and other raw materials at temperatures ranging from 1400 to 1600 °C, resulting in a molten substance. This molten material is rapidly cooled and quenched with water to yield the final product, often discharged as waste slag. Typically, every ton of yellow phosphorus production generates 8 to 10 tons of waste residue. Due to its low utilization rate, significant quantities of yellow phosphorus slag accumulate over large areas, which are vulnerable to rainfall-induced leaching of phosphorus and fluorine into the soil. This contamination affects water sources and impedes plant growth, posing serious environmental risks and threats to human health [11,12,13,14,15]. With the advancement of industrial yellow phosphorus production, the accumulation of yellow phosphorus slag has exacerbated environmental pollution issues. Addressing the storage and utilization of yellow phosphorus slag has thus become a critical challenge for the development of the phosphorus chemical industry.

The industrial applications of yellow phosphorus slag primarily focus on the cement industry on a large scale [16,17,18]. Its uses include serving as raw materials for cement, additives in phosphorus slag cement, low-heat clinker phosphorus slag cement, non-clinker cement, and composites. Additionally, phosphorus slag is utilized in various sectors, such as the production of building materials like phosphorus slag bricks, modified hardeners, glazes, active aggregates, and road base materials, alongside its role in Portland cement production [19,20]. However, its application in water conservancy, hydropower engineering, and the construction industry has been limited due to factors like slow setting, low early strength, and limited admixture compatibility. Currently, both domestic and international research on yellow phosphorus slag primarily focuses on enhancing its performance in concrete, particularly improving early strength [21,22,23,24,25]. In the field of mine filling, only a few mines have explored its potential cementitious properties, finding that grinding yellow phosphorus slag into a superfine powder does not significantly improve its overall cement substitution effect [26].

The utilization of yellow phosphorus slag as a substitute or partial substitute for cement primarily hinges on its potential cementitious properties [27,28,29]. Yellow phosphorus slag is ground into phosphorus slag powder, also known as ultrafine powder, and mixed into filler slurries. However, the slag powder exhibits a retarding effect, leading to low early strength and potential inadequacies in meeting short-term structural requirements in mining stopes. Moreover, the presence of P_2_O_5_ and fluorine in phosphorus slag limits its inclusion in filler slurries. In the production of Portland cement using phosphorus slag as a mixed material, the extended setting time, particularly when the phosphorus slag content is high, with initial setting times exceeding 6 h, significantly hinders its practical application [1,30]. Laboratory tests have indicated that the uniaxial compressive strength of yellow phosphorus slag-phosphogypsum test blocks notably improves with treatment using NaOH or CaO, with optimal contents of 5% and 8%, respectively. However, achieving uniform mixing of NaOH and CaO in practical field applications remains challenging due to their low quantities, contrasting with controlled indoor settings where materials can be evenly blended to function effectively as alkaline stimulants [31,32,33,34]. These limitations contribute to unsatisfactory outcomes in industrial applications.

In general, yellow phosphorus slag has some potential gelling activity and can partially substitute for cement in filling mining. However, due to its retarding effect, it cannot be widely applied and popularized.

The cementing filler is composed of aggregate, gelling agent, and water [35,36,37]. Frequently employed aggregates encompass tailings, river sand (comprising both river and sea sand), gravel, and various abrasives, to name a few. The commonly used gelling agent is ordinary Portland cement. Currently, tailings continue to predominate as the primary mineral filler aggregate, irrespective of the number of mines utilizing tailings for filling or the overall volume of tailings employed. To enhance the strength of the filling material while minimizing cement consumption, the long-standing fundamental approach in mine filling design and research is as follows:

(1) The technical route to improve slurry concentration: from graded tailings cemented filling, full tailings high-concentration cemented filling, and paste filling.
(2) Seeking lower-cost cementing agents, such as adding fly ash, lime, gypsum, etc., or filling with red mud.

The main technical consensus in mine filling is as follows:

(1) When the cement dosage remains constant, the higher the slurry concentration, the greater the strength of the backfill;
(2) Under the strength standard of a backfill, the higher the slurry concentration, the lower the cement dosage and the lower the cost;
(3) Adding coarse aggregates can effectively improve strength.

To summarize, the extensive discharge of yellow phosphorus slag has significantly harmed the environment over time. Attempts to partially replace cement with yellow phosphorus slag have proven ineffective due to its limited availability, failing to address the ecological and environmental issues stemming from its widespread discharge. Therefore, this study explores the use of yellow phosphorus slag as an aggregate in mine filling materials [38]. It examines the grading, density, and compressive strength of a filling material comprising yellow phosphorus slag, tailing sand, and cement, proposing a new approach to utilize yellow phosphorus slag in mine filling applications.

## 2. Model and Application of Filling Aggregate Pile Compactness

Packing compaction is a critical property of solid particle mixtures. In mine-cemented backfill, aggregates constitute 67% to 75% of the backfill volume, making them pivotal in determining backfill strength and cost [39,40,41,42]. The bulk density of aggregates comprehensively reflects their interstitial properties, encompassing porosity, particle size, shape, and gradation relationships. It serves as a crucial parameter for describing the aggregate bulk system. Maximizing aggregate compactness widens the interstitial gaps between backfill aggregates, achieving a tightly interlocked state that optimizes the strength properties of the mixed aggregates.

In this section, we employ the following test schemes as a research methodology: experiment on packing compaction of yellow phosphorus slag and tailing.

### 2.1. The Research Analysis and Evaluation of a Single Aggregate

The physical parameters of tailing selected for this experiment are shown in Table 1.

The grain size distribution of Beidoushan tailings shows characteristics of “fewer coarse particles, fine grains, and a higher proportion of intermediate grain sizes”. The particle size distribution index of the tailings from the mine is 0.5, meeting the grading criteria set forth by Fuller. The particle size distribution of these tailings is favorable, with a high density. However, the tailings exhibit a very fine texture, with particle sizes predominantly below 200 mesh. If tailings are used alone for filling, the excessive presence of fine particles will result in higher cement consumption, which is detrimental to the development of cemented backfill strength. Therefore, although the tailings from the mine have an ideal gradation index, coarse aggregate must be mixed into the filling process to reduce cement consumption and enhance the strength of the filling.

(1) $$P_{x}=100{\left(\frac{d}{D}\right)}^{n}$$

In the formula, n is the gradation decreasing coefficient.

#### 2.1.1. The Analysis and Evaluation of Tailings Gradation

**Figure 1:**
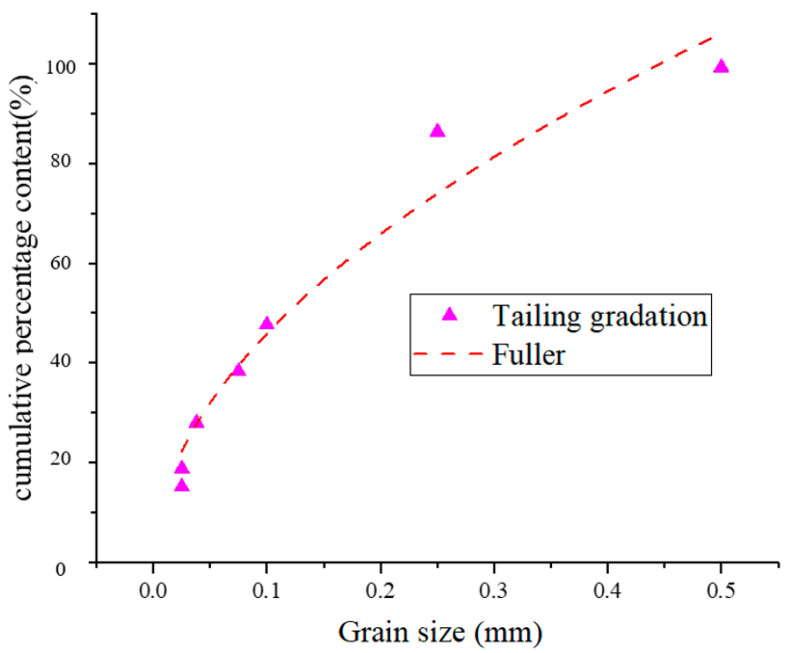
The comparison of grading frequency between tailing and Fuller.

The particle size distribution of Beidou Mountain tailings presents the characteristic of ‘few coarse particles, many fine particles and high proportion of intermediate particle size’. The particle size distribution index of the mine tailings as shown in Figure 1 is 0.5, which meets the grading criteria proposed by Fuller. This distribution is favourable, hence the high density. However, the tailings are very fine textured with particle sizes predominantly below 200 mesh. If the tailings were used alone for filling, the excess fines would lead to an increase in cement consumption, which would be detrimental to the development of cementitious backfill strength. Therefore, although mine tailings have an ideal gradation index, coarse aggregates must be incorporated into the filling process to reduce cement consumption and increase filling strength.

#### 2.1.2. Analysis and Evaluation of Yellow Phosphorus Slag Gradation

**Figure 2:**
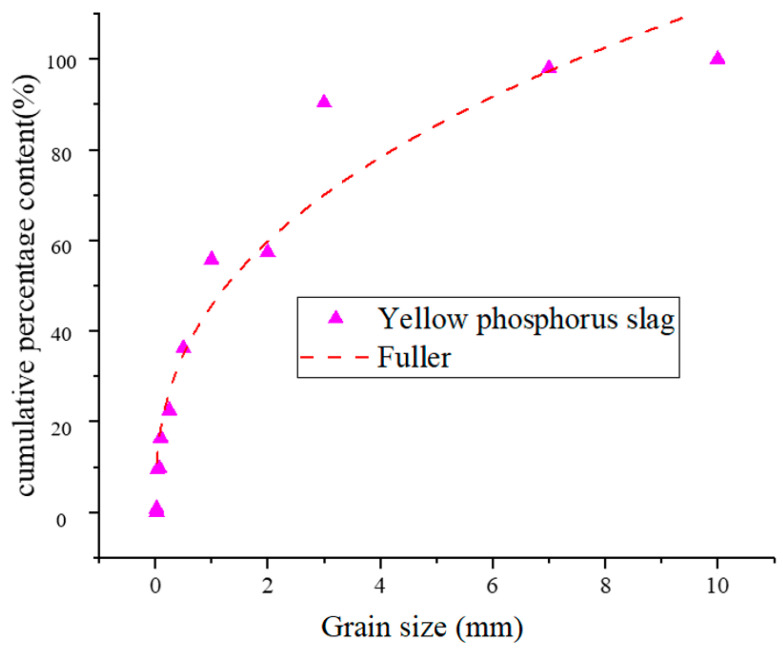
The comparison of grading frequency between yellow phosphorus slag and Fuller.

The comparison of grading frequency between yellow phosphorus slag and Fuller is shown in Figure 2. The gradation curve of yellow phosphorus slag is shown in Figure 2. The gradation index of yellow phosphorus slag is n = 0.97. Evidently, the gradation of yellow phosphorus slag does not conform to Fuller’s gradation requirements. Indeed, the granules of yellow phosphorus slag are excessively large, which can lead to insufficient fine particle content within the slurry and result in compositional irregularities [43,44,45]. Therefore, it is suggested that the yellow phosphorus slag should not be used for filling alone but should be used as the mixed filling material together with coarse aggregate and tailings.

### 2.2. Study on the Compactness of Two Kinds of Aggregate

#### 2.2.1. Space-Filling Effect of Particles

**Figure 3:**
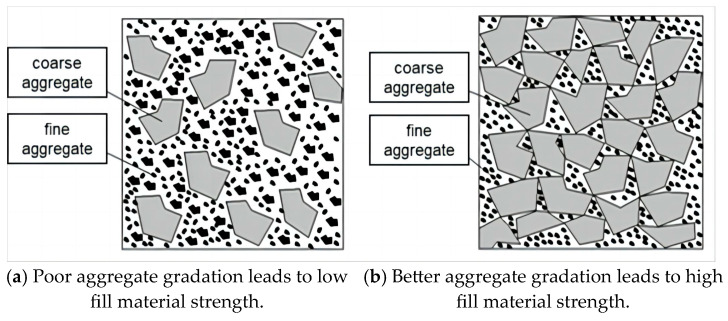
Schematic diagram of aggregate caulking effect (**a**,**b**).

**Figure 4:**
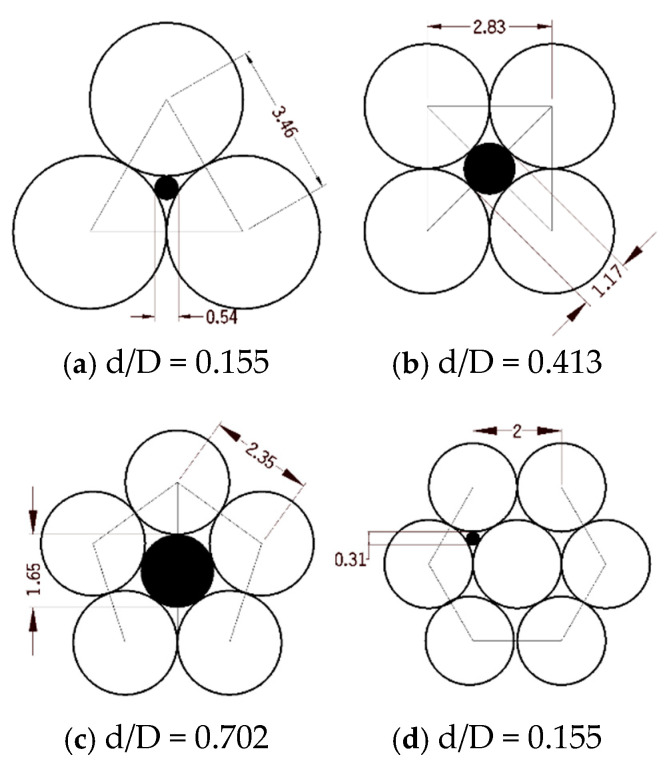
Schematic diagram of the interstitial effect of two aggregates.

When two aggregates of different particle sizes are mixed, the fine aggregate can fill the pores of the coarse aggregate, thereby reducing the pores and increasing compactness. However, one reason for the poor grading effect of the two aggregates is the ‘loose effect’ between the aggregates, preventing the coarse aggregate from forming a skeleton structure [46,47,48,49]. Consequently, the strength of the aggregate cannot be fully utilized; under normal circumstances, this type of aggregate struggles to form a high-strength filling body. If various aggregates are represented by loose particles, the phenomenon of the ‘interstitial effect’ is illustrated in Figure 3. Similarly, if the aggregate is represented by spherical particles, the gap-filling effect is maximized, as shown in Figure 4.

#### 2.2.2. Two Aggregate Compactness Models

From a quantitative analysis standpoint, the compactness of the cemented filling aggregate in the mine (Φ) is defined as the ratio of the solid volume to the total volume of the aggregate system. From a different perspective, the porosity of the aggregate system (ω) signifies the proportion of void volume to the total aggregate system volume. According to the definition of compactness and porosity, Φ = 1 − Ω. The relationship between porosity ratio ε and porosity ω is ε = ω/(1 − ω). It is assumed that the backfill aggregate bulk system is mixed with coarse aggregate 1 and fine aggregate 2, and the parameters of the backfill aggregate bulk system are defined as follows:

(1) x: Ratio of coarse aggregate in two kinds of aggregates, that is, the ratio of the mass of aggregate 1 to the mass of mixed aggregate;
(2) m: Total mass of mixed aggregate, kg;
(3) m_1_: Mass of aggregate 1 (coarse aggregate) of the two mixed aggregates, kg;
(4) m_2_: Mass of aggregate 2 (fine aggregate) in the two mixed aggregates, kg;
(5) k: Mass ratio of aggregate 1 to aggregate 2 in the two mixed aggregates;
(6) ρ: Density of the mixed aggregate, t/m^3^;
(7) $\rho_{1}$: Density of aggregate 1, t/m^3^;
(8) $\rho_{2}$: Density of aggregate 2, t/m^3^;
(9) Φ: Packing density of mixed aggregate
(10) $\Phi_{1}^{*}$: Compactness of aggregate 1 (coarse aggregate) in a certain mixing state;
(11) Φ_1_: Packing density of aggregate 1;
(12) Φ_2_: Packing density of aggregate 2;
(13) ω: Porosity of mixed aggregate;
(14) ω_1_: Porosity of aggregate 1;
(15) ω_2_: Porosity of aggregate 2.

The mathematical model of the compactness of the two aggregate mixtures was established as follows:

According to the meaning of parameters ρ, $\rho_{1}$, $\rho_{2}$, and X, we have the following equations:

(2) 1ρ=xρ1+1−xρ2

As per the property that the respective solid volumes of the two aggregates remain unchanged before and after mixing, the following formula can be established:

(3) $$\frac{m}{\rho \Phi }=\frac{xm}{\rho_{1}\Phi_{1}^{*}}+\frac{(1-x)m}{\rho_{2}\Phi_{2}}$$

According to the significance of the parameter, its value range is 0 < x < 1, and then there are the following cases:

1. Boundary condition 1: When x = 1, that is, when there is no aggregate 2, Equation (3) can be obtained as follows:
2. Boundary condition 2: When x = 0, that is, when there is no aggregate 1, Equation (3) can be obtained:
3. When x is very small, that is, aggregate 2 occupies an absolute dominant proportion, and aggregate 1 cannot form a skeleton structure and its pores are completely filled by aggregate 2. At this time, the porosity of aggregate 1 can be regarded as zero, that is, =0. This situation is shown in Figure 3a; that is, aggregate 1 does not form a skeleton structure in the mixture, but is completely ‘suspended’ in aggregate 2. This state is quantitatively expressed as follows:
  (4) $$x\leq \frac{\rho_{1}\Phi_{1}}{\rho }\;\Phi_{1}^{*}=1-\omega_{1}=1$$
  Substituting Formula (4) into Formula (3) immediately produces:
  (5) $$\frac{m}{\rho \Phi }=\frac{xm}{\rho_{1}}+\frac{(1-x)m}{\rho_{2}\Phi_{2}}\;x\leq \frac{\rho_{1}\Phi_{1}}{\rho }$$
4. When x is large, which means that aggregate 1 occupies an overwhelmingly significant proportion, aggregate 1 forms a complete skeletal structure within the mixture. Meanwhile, aggregate 2 serves solely as a gap filler. Aggregate 2 interstitially fills the pores of aggregate 1 from individual components to the entirety, as illustrated in Figure 3b. This state is quantitatively expressed as follows:
  $$\omega_{1}^{*}=\omega_{1}-(1-x\frac{\rho }{\rho_{1}})=1-\Phi_{1}-(1-x\frac{\rho }{\rho_{1}})=x\frac{\rho }{\rho_{1}}-\Phi_{1}\;\Phi_{1}^{*}=1-\omega_{1}^{*}=\Phi_{1}+(1-x\frac{\rho }{\rho_{1}})$$
  (6) $$\mathrm{Among}\;\mathrm{them}:\;x>\frac{\rho_{1}\Phi_{1}}{\rho }$$

In summary, the stacking compactness model functions of the two mixtures can be established as follows:

(7) $$\Phi =\left\{\begin{array}{l} \frac{1}{\rho }\cdot {\left(\frac{x}{\rho_{1}}+\frac{1-x}{\rho_{2}\Phi_{2}}\right)}^{-1}\;x\leq \frac{\rho_{1}\Phi_{1}}{\rho }\cdot \cdot \cdot \cdot \cdot \cdot \cdot \cdot \cdot \cdot \cdot \cdot \cdot \cdot \cdot \cdot \cdot \cdot \cdot \cdot \cdot \cdot ()\\ \frac{1}{\rho }\cdot {\left[\frac{x}{\rho_{1}\left(\Phi_{1}+1-x\frac{\rho }{\rho_{1}}\right)}+\frac{1-x}{\rho_{2}\Phi_{2}}\right]}^{-1}\;x>\frac{\rho_{1}\Phi_{1}}{\rho }\cdot \cdot \cdot \cdot \cdot \cdot \cdot \cdot ()\\ \rho =x\rho_{1}+\left(1-x\right)\rho_{2}\;0\leq x\leq 1\cdot \cdot \cdot \cdot \cdot \cdot \cdot \cdot \cdot \cdot \cdot \cdot \cdot \cdot \cdot \cdot \cdot \cdot \cdot \cdot \cdot () \end{array}\right.$$

In the formula, the symbolic meaning is the same as before.

### 2.3. Application of Two Aggregate Compactness Models

It can be seen from the two kinds of mixture compaction model functions that the mixed compactness of the two aggregates is a single-valued function of the ratio of the two mixed aggregates. In the mixed aggregate bulk system, the pile compactness $\Phi_{1}$ of coarse aggregate 1, the pile compactness $\Phi_{2}$ of fine aggregate 2, the density ρ of the two mixed aggregates, the density $\rho_{1}$ of coarse aggregate 1, the density $\rho_{2}$ of fine aggregate 2, etc. can be measured by experiment. It is noteworthy that all these parameters are subject to experimental measurement. All can be measured through experiments.

#### Experimental Analysis of the Compactness of Yellow Phosphorus Slag and Tailings

The compactness of piles formed by mixing yellow phosphorus slag and tailings follows a specific pattern: when the proportion of crushed waste rock aggregate is below 0.6, the compactness of the mixture increases as the amount of coarse waste rock particles increases. As the quantity of crushed waste rock aggregate rises, so does the number of coarse particles. This phenomenon enhances the ‘skeleton’ effect, leading to an overall increase in the compactness of the mixture. However, as the amount of coarse waste rock aggregate continues to increase, the amount of tailing sand gradually decreases. Concurrently, the pile density of the mixture also shows a decreasing trend.

**Figure 5:**
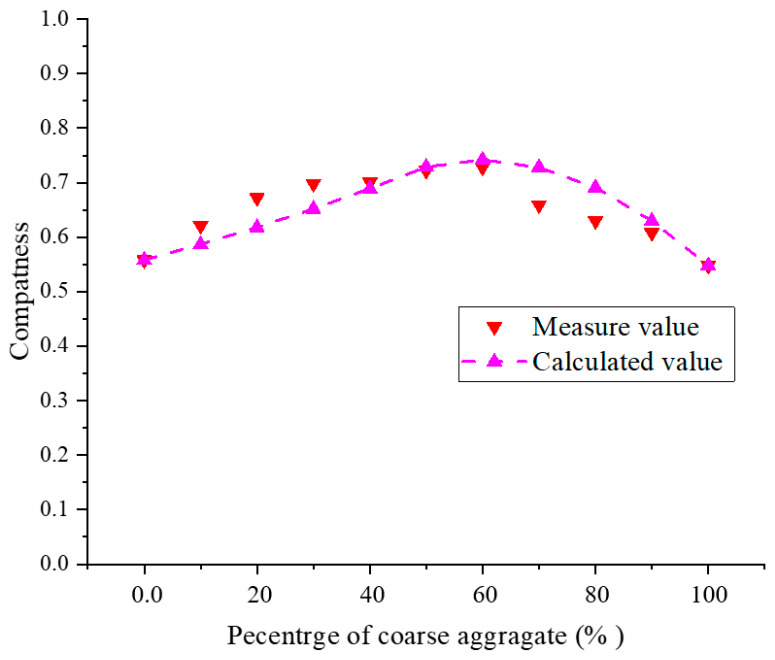
Comparison between the measured and calculated values of the stacked compactness of yellow phosphorus slag and tailing.

Figure 5 illustrates that the calculated results for the packing compactness of the yellow phosphorus slag and tailings mixture align with the measured results. The findings indicate that the packing compactness reaches its maximum when the coarse aggregate comprises 60%, with a measured compactness of 0.73 at this point. Beyond this, the compactness begins to decrease. In the mixed filling, the porosity of the aggregate decreases gradually as the amount of yellow phosphorus slag increases, reaching its minimum when the yellow phosphorus slag accounts for 60%. The term ‘maximum packing density’ refers to the condition in which the filling slurry exhibits its highest cementation strength under the same conditions.

### 2.4. Verification of Mixing of Two Aggregates Based on Fuller’s Grading Theory

The two aggregates were mixed in accordance with their recommended and optimal ratios based on the results of the packing compactness test. The gradation that resulted from the mixing of the two fillers was then examined using the Fuller gradation theory to further confirm whether the filling mixture with this ratio has the highest level of compactness and whether it satisfies the requirements for pipeline transportation.

#### 2.4.1. Gradation Analysis After Mixing of Yellow Phosphorus Slag and Tailings

**Figure 6:**
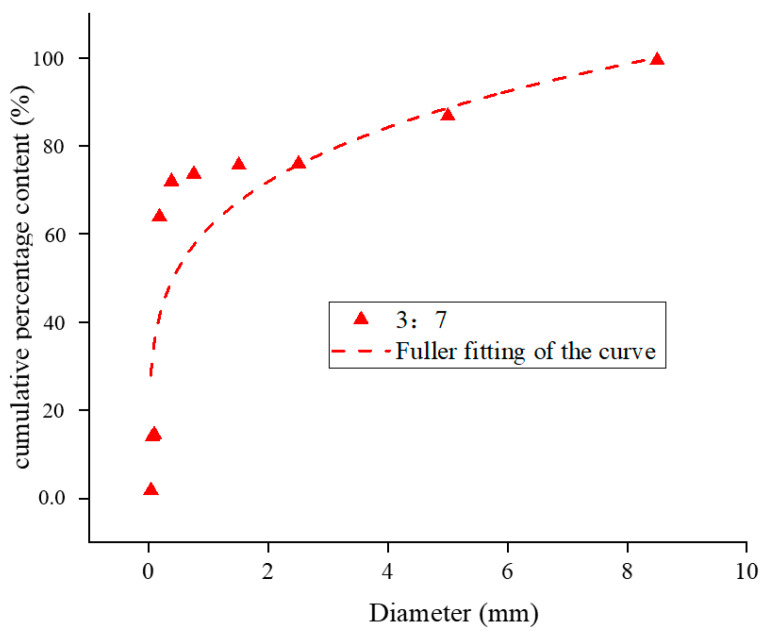
Cumulative percentage content particle size distribution curve of yellow phosphorus slag tailings 3:7 mixture.

**Figure 7:**
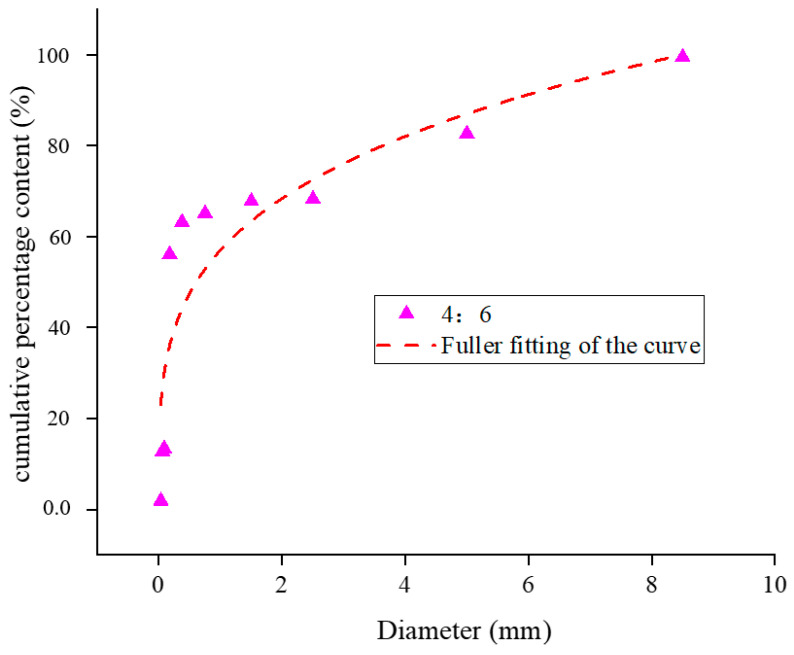
Cumulative percentage content particle size distribution curve of yellow phosphorus slag tailings 4:6 mixture.

**Figure 8:**
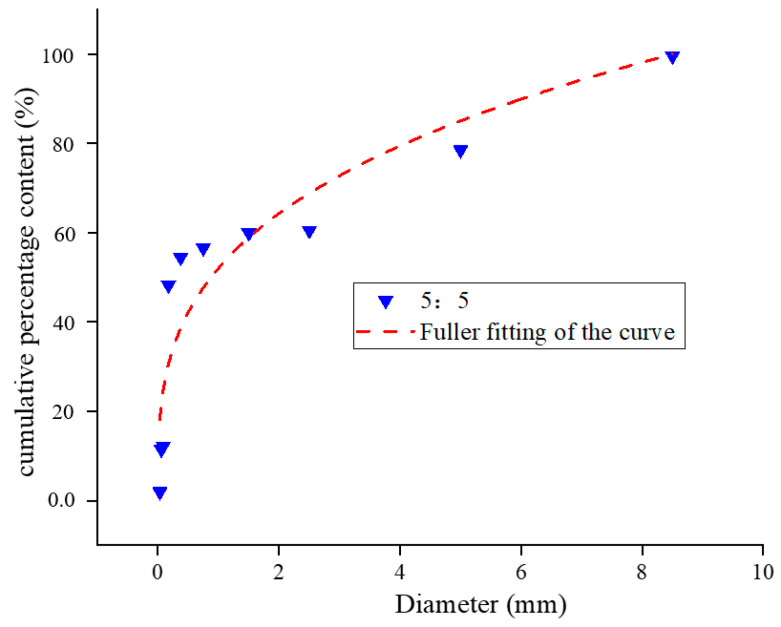
Cumulative percentage content particle size distribution curve of yellow phosphorus slag tailing 5:5 mixture.

Yellow phosphorus slag and tailing were mixed at 3:7, 4:6, and 5:5. The Fuller fitting curves after mixing in different proportions are shown in Figure 6, Figure 7 and Figure 8.

For large particle objects, since the particles are large, their contact area is also relatively large, so during the filling process, the contact force between the particles is stronger and the interaction is more complex. At the same time, since the surface area of the large particle object is relatively small, its surface can be covered by other particles during the filling process, which leads to a better filling effect. In addition, large particles are more likely to roll and slide during the filling process, which can lead to more voids and gaps in the filling process.

For small particle objects, because the particles are small, their contact area is also relatively small, so in the filling process, the contact force between the particles is weak and the interaction is relatively simple. At the same time, because the surface area of the small particle object is relatively large, its surface cannot be covered by other particles during the filling process, which results in a relatively poor filling effect. In addition, small particles are more prone to rolling and sliding during the filling process, which results in more voids and gaps in the filling process.

The difference in the mechanism of filling large and small particles lies mainly in the contact force between particles and the state of motion between particles, which will affect the filling effect and filling quality. Therefore, it is necessary to choose appropriate methods and techniques according to the characteristics of different particles in order to obtain better filling effect and higher filling quality when performing filling operations.

The R^2^ value is a widely used metric to assess the degree to which a regression model explains variability in the data, ranging from 0 to 1. Higher values indicate a stronger fit, with 1 representing a perfect fit and 0 indicating no explanatory power. While the interpretation of R^2^ values can vary across different disciplines and research contexts, general guidelines suggest that an R^2^ above 0.7 indicates a strong model fit, values between 0.4 and 0.7 denote a moderate fit, and values below 0.4 suggest a weak fit. According to the gradation distribution curve of yellow phosphorus slag and tailings mixtures, the particle size distribution aligns well with the Fuller equation at the recommended mixing ratios. The mixtures with ratios of 3:7, 4:6, and 5:5 exhibit fitting indices of 0.23, 0.26, and 0.30, respectively, with corresponding R^2^ values of 0.73, 0.80, and 0.85. Among these, the 5:5 ratio is closest to Fuller’s curve, indicating superior packing density, lower porosity, and enhanced cementing strength under identical conditions.

#### 2.4.2. Assessment of Fuller’s Precision

To verify the accuracy of Fuller gradation, the mean absolute error (MAE) and mean absolute percentage error (MAPE) were selected to test the fitting results.

Mean absolute error (MAE) is another metric used to measure the accuracy of predictions. It calculates the average of the absolute values of the differences (or errors) between the predicted values and the actual values. Unlike MAPE (mean absolute percentage error), MAE does not convert the errors into a percentage form but directly represents the size of the average error in numerical form. When the calculated result value is relatively small, the data are considered reliable.

(8) $$MAE=\frac{1}{n}\sum_{i=1}^{n}\left|y_{i}-y\right|$$

(1) y_i_ represents the actual value;
(2) y represents the fitted value;
(3) n is the number of observations.

Mean absolute percentage error (MAPE) is a metric used to measure the accuracy of predictions. It calculates the absolute value of the difference between the predicted value and the actual value, converts it into a percentage form, and represents the size of the average error. MAPE provides an intuitive measure of error rate, helping to evaluate the accuracy of prediction models. Generally speaking, a MAPE value of less than 10% may be considered good predictive performance.

(9) $$MAPE=\frac{100\%}{n}\sum_{i=1}^{n}\left|\frac{y_{i}-y}{y_{i}}\right|$$

(1) y_i_ represents the actual value;
(2) y represents the fitted value;
(3) n is the number of observations.

The grading frequency comparison of tailings and Fuller’s ore by applying MAE and MAPE in Table 2, Comparison of grading frequency of yellow phosphorus slag and Fuller’s slurry, and three comparisons of the ratio of yellow phosphorus slag to tailings can be tested to show that the results of this monitoring are reliable. 

## 3. Research on Compressive Strength of Cemented Backfill

### 3.1. Experiment Scheme Design

To more accurately study the relationship between the strength of filling slurry at different proportions and the consumption of filling materials, a comprehensive test method was adopted for the strength test. The filling strength test scheme in Table 3 was designed to analyze the strength of the cementing body and its multifactor influence relationship under the mixture ratio of yellow phosphorus slag and tailings.

According to the technical standard for filling, specimens were measured using a steel mold of dimensions 100 mm × 100 mm × 100 mm and weighed using an electronic scale. After disassembly, the test blocks were subjected to standard curing in a controlled environment with a temperature of 28 °C and humidity of 85%.

We conducted strength experiments based on Table 3, and the experimental results can be found in Appendix A.

### 3.2. Cementing Strength Model and Data Analysis

#### 3.2.1. Multi-Factor Nonlinear Model

For many years, research has primarily centered on studying the influence of the water–cement ratio on the strength of filling materials, or the effects of cement addition and porosity on strength. As a result, the practical application of resulting empirical formulas has been quite limited and does not fully encompass the broader influence of multiple factors on the strength of filling materials. According to this analysis, it is evident that:

(1) The strength of the cemented body has an inverse exponential relationship with the slurry water–cement ratio.
(2) The strength of the test block increases significantly with the densification of the aggregate packing.
(3) The strength of the test block increases significantly with the increase in slurry volume concentration.
(4) The strength of the test block increases significantly with the increase in cement addition.

Combining these findings with previous research results, a new model of cemented body strength is proposed:

(10) $$R=k\times R_{c}\times \Phi \times e^{C_{v}}\times {\left(\frac{W}{C}\right)}^{a}$$

Under the condition of slurry saturation (minimum moisture content condition):

(11) $$\frac{V_{W-C}}{1-\Phi }\geq 1$$

(1) R—Compressive strength of cement, MPa;
(2) Φ—Aggregate pile compactness;
(3) $V_{W-C}$—Volume of cement paste per unit volume of slurry, m^3^;
(4) $C_{v}$—Volume concentration of the slurry;
(5) W/C—Slurry water–cement ratio;
(6) *R*_c_—Cement grade, MPa;
(7) a, k—experimental constant.

The advantage of this model is that it comprehensively considers the influence of aggregate, cement, and water on strength, and it is conducive to regression analysis.

#### 3.2.2. Regression Statistical Method of Strength Model

On the basis of the strength prediction model, the experimental data is processed using multiple nonlinear regression analysis. The regression analysis model and process are as follows:

Suppose that the outcome of the test is the dependent variable y, the influences are the independent variables x, and there are m independent variables, denoted as x_1_, x_2_, …, x_i_, …, x_m_, and suppose that the test measures n sets of data:

(x_11_, x_21_, …, x_i1_…, x_m1_, y_1_) (x_12_, x_22_, …, x_i2_…, x_m2_, y_2_) (x_1j_, x_2j_, …, x_ij_…, x_mj_, y_j_) (x_1n_, x_2n_, …, x_in_…, x_mn_, y_n_)

Then, the multiple linear regression equation can be expressed as:

(12) $$\hat{y}=b_{0}+b_{1}x_{1}+b_{2}x_{2}+\ldots +b_{m}x_{m}$$

In this formula, b_0_ is a constant, and b_i_ is the partial regression coefficient of the experimental result for the influencing factor x_i_.

According to the principle of the least squares method, to minimize the residual sum of squares of the multiple regression equation, b_0_ and b_i_ (i = 1, 2, …, m) can be obtained. The residual sum of Q_e_ can be expressed as:

(13) $$Qe=\sum_{j=1}^{n}(y_{i}-{\hat{y}}_{i}{)}^{2}=\sum_{j=1}^{n}[(y_{i}-(b_{0}+b_{1}x_{1j}+b_{2}x_{2j}+\ldots +b_{m}x_{mj}){]}^{2}$$

Taking partial derivatives of the residual sum of squares with respect to b_0_ and b_i_:

(14) $$\frac{\partial Qe}{\partial b_{0}}=-2\sum_{j=1}^{n}[(y_{i}-(b_{0}+b_{1}x_{1j}+b_{2}x_{2j}+\ldots +b_{m}x_{mj}){]}^{2}=0$$

(15) $$\frac{\partial Qe}{\partial b_{i}}=-2\sum_{j=1}^{n}x_{ij}[(y_{i}-(b_{0}+b_{1}x_{1j}+b_{2}x_{2j}+\ldots +b_{m}x_{mj})]=0$$

In this formula, j = 1, 2, …, m; these equations form a system of m + 1 equations:

(16) $$\left\{\begin{array}{l} nb_{0}+(\sum_{j=1}^{n}x_{1j})b_{1}+(\sum_{j=1}^{n}x_{2j})b_{2}+\cdots +(\sum_{j=1}^{n}x_{mj})b_{m}=\sum_{j=1}^{n}y_{i}\\ b_{0}(\sum_{j=1}^{n}x_{1j})+(\sum_{j=1}^{n}{x^{2}}_{1j})b_{1}+(\sum_{j=1}^{n}x_{1j}x_{2j})b_{2}+\cdots +(\sum_{j=1}^{n}x_{1j}x_{mj})b_{m}=\sum_{j=1}^{n}x_{1j}y_{i}\\ b_{0}(\sum_{j=1}^{n}x_{2j})+(\sum_{j=1}^{n}x_{2j}x_{1j})b_{1}+(\sum_{j=1}^{n}{x^{2}}_{2j})b_{2}+\cdots +(\sum_{j=1}^{n}x_{2j}x_{mj})b_{m}=\sum_{j=1}^{n}x_{2j}y_{i}\\ \cdots \cdots \\ b_{0}(\sum_{j=1}^{n}x_{mj})+(\sum_{j=1}^{n}x_{mj}x_{1j})b_{1}+(\sum_{j=1}^{n}x_{mj}x_{2j})b_{2}+\cdots +(\sum_{j=1}^{n}{x^{2}}_{mj})b_{m}=\sum_{j=1}^{n}x_{mj}y_{i} \end{array}\right.$$

Solving this system of equations yields b_0_ and b_i_ (i = 1, 2, …, m).

If the relationship between the experimental results and influencing factors is a power function, the model can be set as:

(17) $$Y=AX_{1}^{B}X_{2}^{C}X_{3}^{D}$$

(18) $$\ln Y=\ln A+B\ln X_{1}+C\ln X_{2}+D\ln X_{3}$$

Set up: y = lnY, $b_{0}$ = lnA, $x_{1}$ = $\ln X_{1}$, $x_{2}$ = $\ln X_{2}$, $x_{3}$ = $\ln X_{3}$, $b_{1}$ = B, $b_{2}$ = C, $b_{3}$ = D, then the nonlinear model can be transformed into a linear regression model:

(19) $$y=b_{0}+b_{1}x_{1}+b_{2}x_{2}+b_{3}x_{3}$$

Significance test of regression coefficients and equations:

The sum of the squares of the total deviations from the regression equation Q=$\sum_{j=1}^{n}(y_{i}-\bar{y}{)}^{2}$ degrees of freedom f = n – 1.

Regression sum of squares: $Q_{x}=\sum_{j=1}^{n}(\hat{y}-\bar{y}{)}^{2}$ degrees of freedom f = m. It is due to the change in x. It reflects the change in y due to the linear relationship between x and y and is the part of the equation explained by the regression equation.

Sum of squared residuals: $Q_{e}=\sum_{j=1}^{n}(y_{i}-\hat{y}{)}^{2}$ degrees of freedom f = n − m − 1; this is caused by the observation not falling on the regression surface.

In evaluating the quality of the regression equation, the explanatory power of the equation is often mentioned. This refers to the extent to which the obtained regression equation explains the changes in the dependent variable or how well the equation fits the experimental data. The main parameters are:

Coefficients of determination for equations; R^2^ indicates the ratio of the regression sum of squares to the total sum of squares:

(20) $$R^{2}=\frac{\sum {\left(\hat{y}-\bar{y}\right)}^{2}}{\sum {\left(y-\bar{y}\right)}^{2}}$$

R^2^ takes values between 0 and 1. Values of R^2^ closer to 1 indicate that the variables in the equation explain y better. When the variables in the regression model are linear, R^2^ is a measure of the goodness of fit of the equation. The larger R^2^ is, the better the regression equation fits the data, or the stronger the x–y linear relationship, i.e., the more the independent variables in the regression equation explain y. When R^2^ is equal to 1, all observations fall on the plane of fit. A smaller R^2^ indicates a weaker x–y linear relationship, a stronger independence between them, or that knowledge of x does not contribute to the prediction of y. As R^2^ approaches 0, it indicates that x and y have little or no linear relationship, but there may be a strong nonlinear relationship.

Complex correlation coefficient MR: Square the multivariate correlation coefficient R^2^ to obtain the multivariate correlation coefficient MR.

R has a value range of [0, 1] and is a measure of the degree of the multivariate linear relationship between y and x. The closer the value is to 1, the closer the linear relationship between y and x is. When R = 1, all observations fall on the fitting plane; when equal to 0, the linear change in y is independent of the change in X. The value of 1 is a measure of the degree of linear relationship between y and x.

Adjusted coefficient of determination:

$${R^{2}}_{adj}:\;{R^{2}}_{adj}=R^{2}-\left[\frac{k}{n-k-1}\left(1-R^{2}\right)\right]=1-\frac{n-1}{n-k-1}\left(1-R^{2}\right)$$

As the number of independent variables increases, the sum of squares of the residuals ${\sum \left(\hat{y}-\bar{y}\right)}^{2}$ decreases, and the coefficient of determination R^2^ increases, even though there are some independent variables that do not have a significant linear relationship with y. Introducing them into the equation causes R^2^ to increase. Therefore, R^2^ is a coefficient affected by the ratio of the number of independent variables to the sample size, and the general convention is that (1:10) or more is good. When this ratio is less than 1:5, R^2^ tends to overestimate the actual goodness of fit. To avoid this situation, the adjusted coefficient of determination ${R^{2}}_{adj}$ is often used in place of R^2^.

As the number of independent variables increases, the sum of squares of the residuals ${\sum \left(\hat{y}-\bar{y}\right)}^{2}$ decreases, and the coefficient of determination R^2^ increases, even though there are some independent variables that do not have a significant linear relationship with y. Introducing them into the equation causes R^2^ to increase. Therefore, R^2^ is a coefficient affected by the ratio (1:10) of the number of independent variables to the sample size, and the general rule is that 1:10 or more is good. When this ratio is less than 1:5, R^2^ tends to overestimate the actual goodness of fit. To avoid this situation, an adjusted coefficient of determination,${R^{2}}_{adj}$, is often used in place of R^2^.

As k increases, the effect of an increase in $\frac{n-1}{n-k-1}$ may be greater than the effect of a decrease in $R^{2}$, thus making RR smaller, and thus RR recognizes the effect of the number of independent variables. ${R^{2}}_{adj}$ will be much smaller than $R^{2}$ when k is close to being the same as n, and ${R^{2}}_{adj}\approx R^{2}$ when n is much larger than k. This better describes the strengths and weaknesses of the explanatory power of the regression equation.

The significance of the regression equation can be tested by the F-test:

$$F=\left(\frac{Q_{x}}{m})/(\frac{Q_{e}}{n-m-1}\right)$$

When the F-value ≧ Fe(m, n − m − 1), then the regression equation is significant at the significance level α, as follows:

When $F\geq F_{0.01}(m,n-m-1)$, the regression equation is highly significant;

When $F_{0.01}(m,n-m-1)\geq F\geq F_{0.05}(m,n-m-1)$, the regression equation is significant;

When $F_{0.05}(m,n-m-1)\geq F\geq F_{0.1}(m,n-m-1)$, the regression equation is significant at the 0.1 level;

When $F<F_{0.1}(m,n-m-1)$, the regression equation is not significant.

The cement strength is not only related to the concentration but also the water–cement ratio and the packing density of the aggregate, so the prediction model of the cement strength of the backfill is established, and its form is as follows:

$$y=42.5\times k\times \phi \times e^{x1}\times {x2}^{a}$$

Among them: y—Uniaxial compressive strength of cemented body (MPa); Φ—aggregate compactness; x1—Slurry volume concentration; x2—water-cement ratio; k, a—regression coefficients.

After establishing the prediction model, we analysed the cementation strength of cemented filling slurries with different ratios at different ages, and the results are shown in Table 4. According to the requirements of design calculation, the strength of cemented strut is above 0.67 MPa.

Yellow phosphorus slag tailing can meet the cementitious strength requirements at 1/6 and 1/7 ash–sand ratios. From the perspective of optimizing cement strength, reducing the cement content can lead to more significant cost savings. In terms of filling cost distribution, the unit price of yellow phosphorus slag is higher than that of waste rock. Therefore, it is recommended to use a mixture of waste rock and tailing sand at ratios of 5:5 and 4:6 in industrial tests, with a 71% mass concentration of cement filling slurry and an ash–sand ratio of 1/7.

## 4. Analysis of Numerical Computation

**Figure 9:**
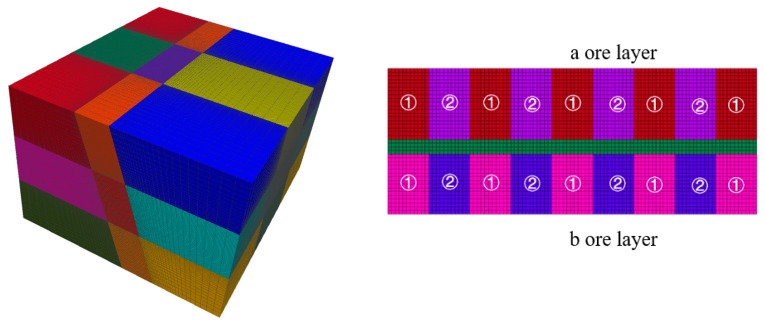
Numerical calculation simulation and schematic diagram of the excavation area.

A geometric model was developed according to the mining methods associated with phosphate deposits. The stage height of the modeled mining site is 60 m, as required by the mining method. The vertical height of each stage is divided into segments every 15 m. In the a and b horizons, the width of the chamber was set at 11 m, and the length of the chamber was equal to the thickness of the a and b horizons. The geometric model is shown in Figure 9. In order to simulate the actual situation, the model was constructed strictly to the actual dimensions of the mining area while also taking into account the effects of mining activities. The rocks surrounding the simulated mining area were also included in the modeling. The final geometric model dimensions were determined to be 299 m by 259 m by 180 m and consisted mainly of overhanging and footwall rock, a-layer ore, b-layer ore, and interburden. The rock mechanical parameters are shown in Table 5.

Taking the a and b ore layers as examples, the excavation calculations for the entire middle section were performed. With an excavation height of 60 m, the calculation results for the simulated region are as follows.

### 4.1. Stress Analysis

**Figure 10:**
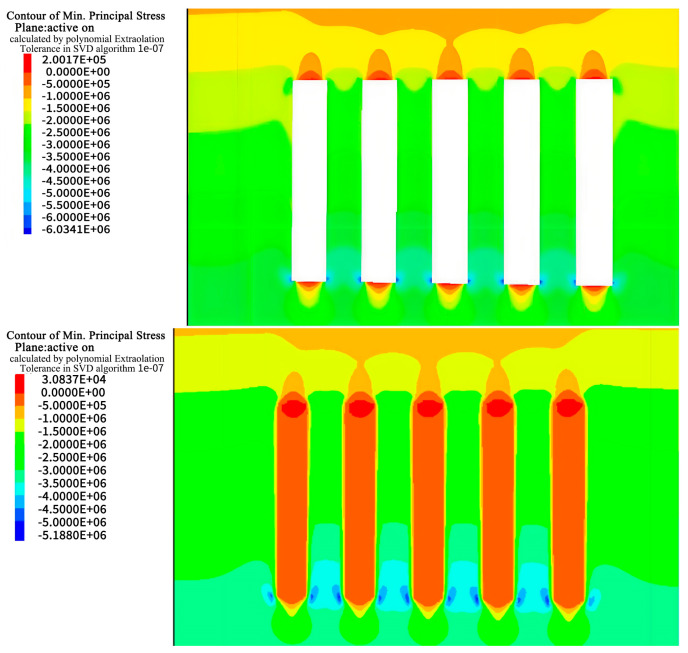
Stress cloud of excavation and filling of Room 1 in the deposit b ore layer.

**Figure 11:**
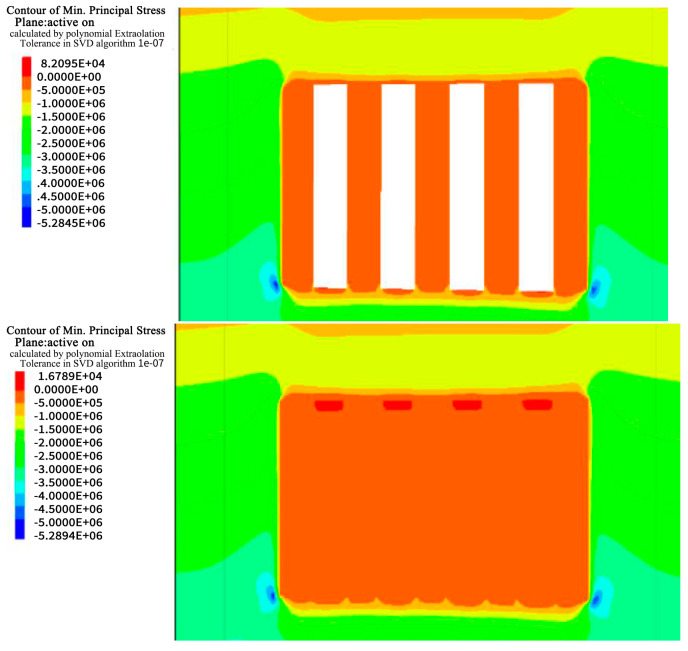
Stress cloud of excavation and filling of Room 2 in the deposit b ore layer.

**Figure 12:**
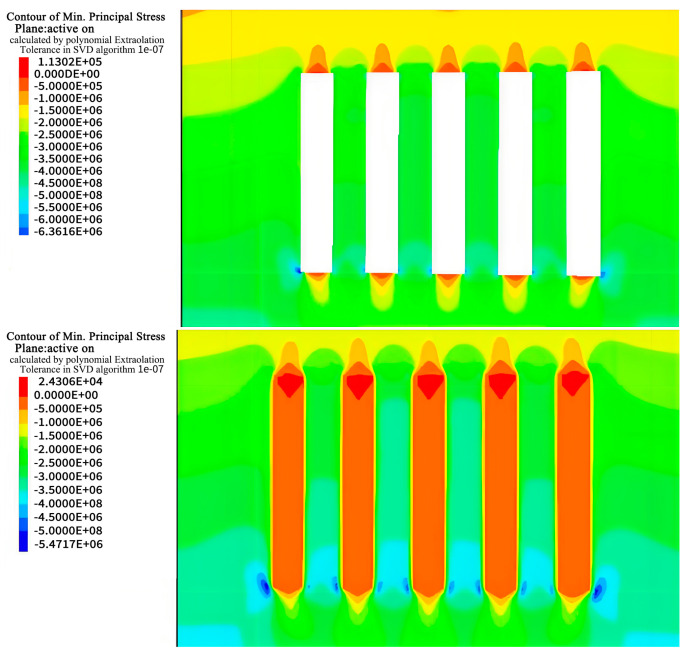
Stress cloud of excavation and filling of Room 1 in the deposit a ore layer.

**Figure 13:**
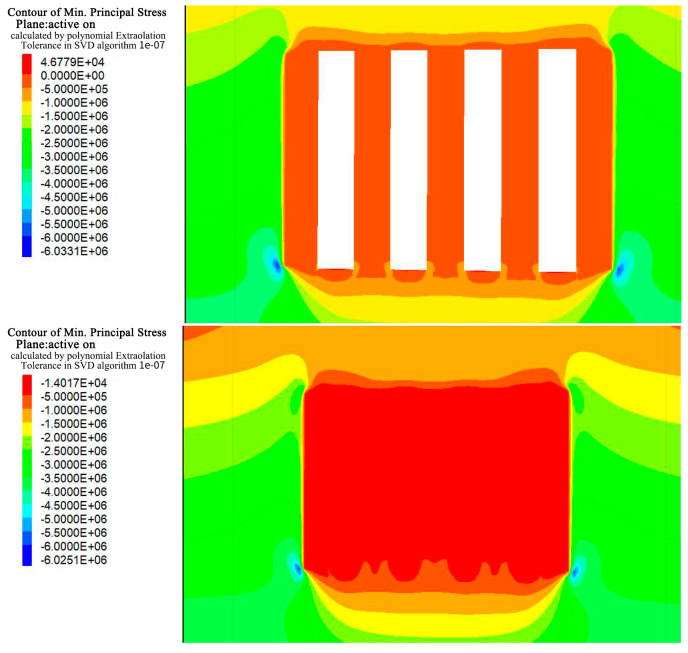
Stress cloud of excavation and filling of Room 2 in the deposit a ore layer.

During excavation of the a and b horizons in Hall 1, the stress field in the rock was disrupted, resulting in areas of stress concentration, especially in the bottom quadrangle, of approximately 6 MPa and 6.3 MPa as shown in Figure 10, Figure 11, Figure 12 and Figure 13. After backfilling, the cemented fill acted as a support structure and relieved the stress concentrations in the bottom corners, reducing the maximum values to 5.2 MPa and 6.0 MPa, indicating that the fill acted as a support.

As excavation proceeded to Room 2 in the a and b ore layers, the cemented fill was exposed on both sides, challenging its self-supporting ability and stability. Research has shown that the cemented fill can effectively mitigate the stress concentration on the roof and floor of the goaf, enabling safe mining operations. Ultimately, after backfilling, the cemented fill formed a stable stress-support structure, providing a favorable stress environment for the safe extraction in the subsequent phase.

### 4.2. Displacement Analysis

**Figure 14:**
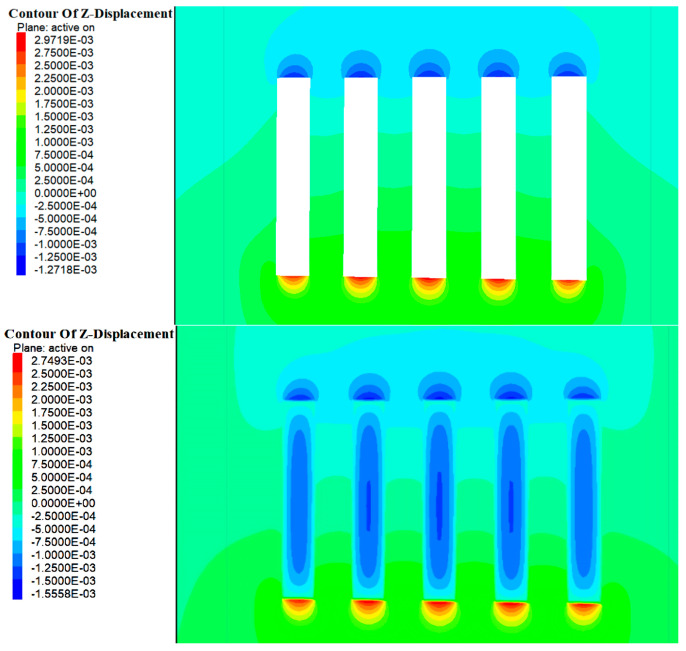
Cloud map of excavation and filling displacements of Room 1 in the deposit b ore layer.

**Figure 15:**
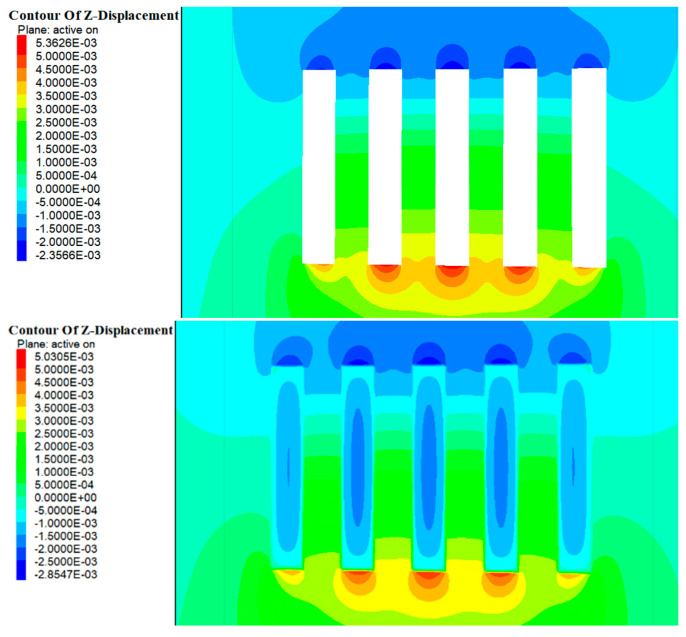
Cloud map of excavation and filling displacements of Room 1 of in the deposit a ore layer.

As shown in Figure 14 and Figure 15, during the excavation of Room 1 in the a and b ore layers, the entire rock mass in this area was exposed, disrupting the original stress field. During the stress redistribution process, displacement zones appeared in the roof and floor of the stope. The maximum vertical displacement at the roof of the b ore layer was approximately 1.2 mm, while the maximum vertical displacement at the floor was about 2.9 mm. For the a ore layer, the maximum vertical displacement at the roof was about 2.3 mm, and at the floor, it was about 5.3 mm. The deformation direction was mainly manifested as roof subsidence and floor uplift. With the introduction of the cemented fill, the deformation of the roof and floor of the stope did not significantly decrease. This was primarily due to the elastic modulus of the cemented fill being smaller compared to the original rock, rendering it less effective in resisting the deformation of the roof and floor.

**Figure 16:**
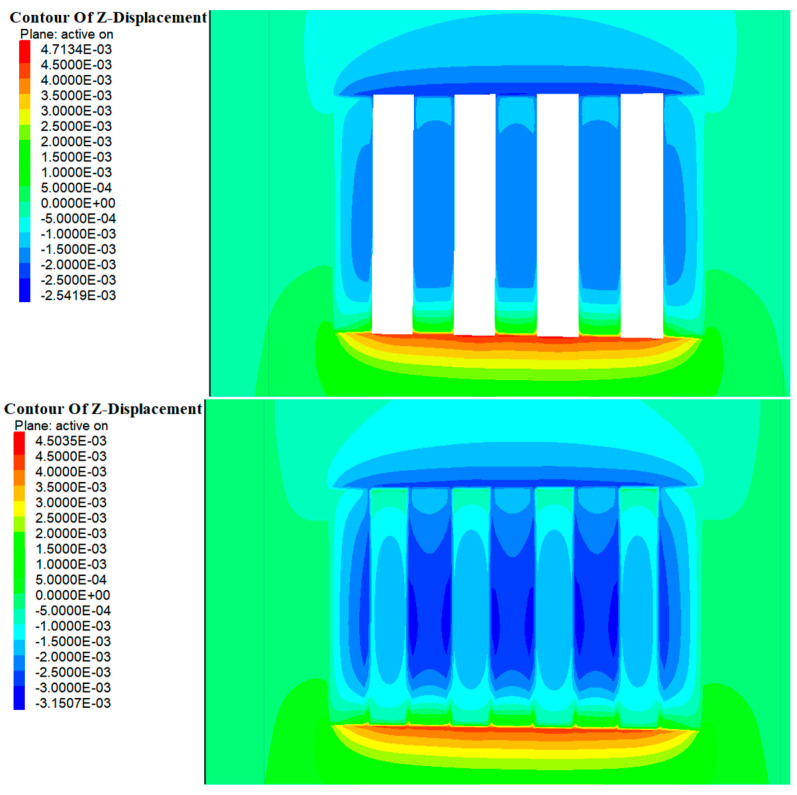
Cloud map of excavation and filling displacements of Room 2 in the deposit b ore layer.

**Figure 17:**
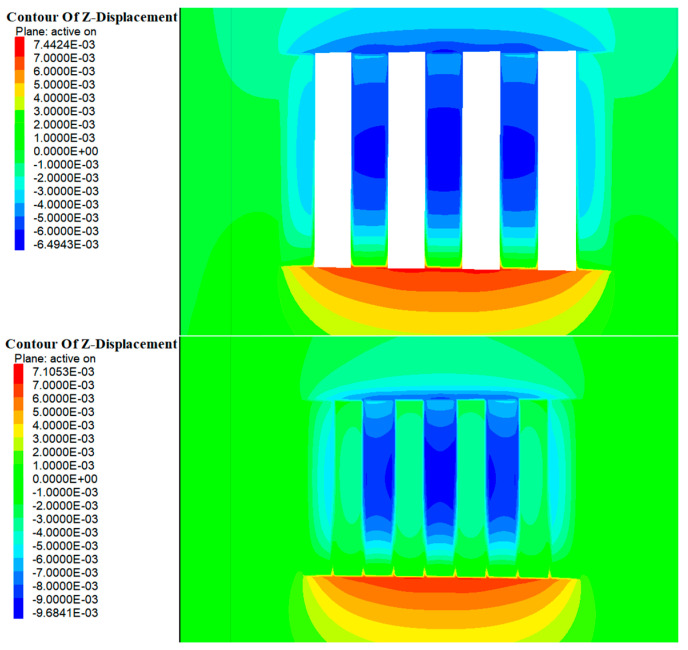
Cloud map of excavation and filling displacements of Room 2 in the deposit a ore layer.

As shown in Figure 16 and Figure 17, with the further excavation of Room 2 in the a and b ore layers, the surrounding rock mass continued to exhibit vertical deformation in the form of roof subsidence and floor uplift. However, unlike the excavation of Room 1, when the cemented pillars acted as a support structure to provide a mining environment for Room 2, their lower elastic modulus meant their deformation resistance was weaker compared with the surrounding rock. Consequently, the range of deformation in the roof continued to increase. At this stage, the maximum vertical displacement at the roof of the b ore layer was approximately 2.5 mm, while the maximum vertical displacement at the floor was about 4.7 mm. For the a ore layer, the maximum vertical displacement at the roof was around 2.3 mm, and at the floor, it was about 5.3 mm.

Upon the completion of the backfilling of Room 2 in the b ore layer, the cemented fill formed an effective and stable stress support structure within the mining area of the b ore layer. At this time, the cemented fill in Room 1 had been under stress for a longer period, evolving from an initial empty unit to a compacted state. Numerical calculations indicated relatively minor displacement changes, suggesting that stress had been transmitted through the cemented fill to the surrounding rock. By contrast, the fill in Room 2 was still in a compressed state and experienced relatively larger displacements, indicating that it absorbed a substantial amount of energy during this period.

### 4.3. Plastic Zone Analysis

**Figure 18:**
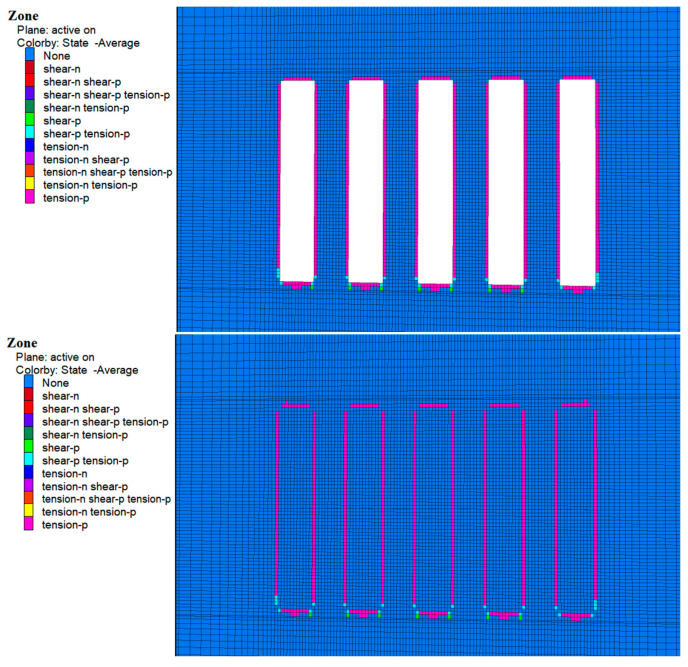
Cloud map of the 1-step mine room excavation and filling of the plastic zone in the b ore layer deposits.

**Figure 19:**
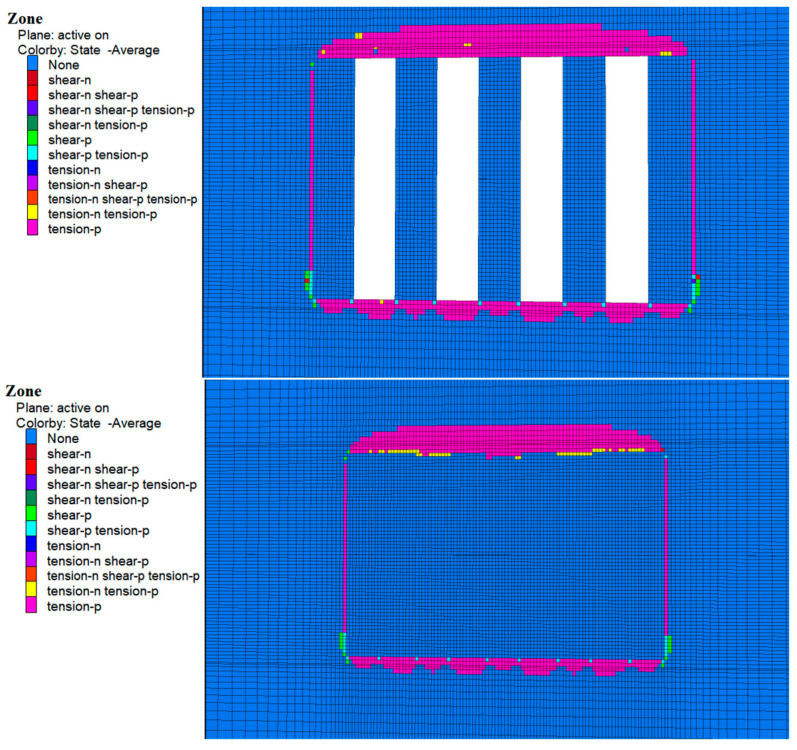
Cloud map of the two-step mine room excavation and filling of the plastic zone in the a ore layer deposits.

During the excavation of Room 1 of the a and b horizons, zones of plastic damage dominated by tensile damage were observed around the perimeter rock as shown in Figure 18 and Figure 19, which were mainly concentrated in the top slab, bottom slab, and sidewalls of the perimeter rock. This damage was mainly due to subsidence of the top slab and bulging of the bottom slab caused by stress release. In addition, a small amount of shear and tensile-shear damage occurred at the bottom corners, which is consistent with the previously mentioned stress concentration in these areas. Although zones of plasticity occur around the entire bed, the damage does not extend to the original ore pillars on either side, suggesting that the environment remained stable during excavation.

**Figure 20:**
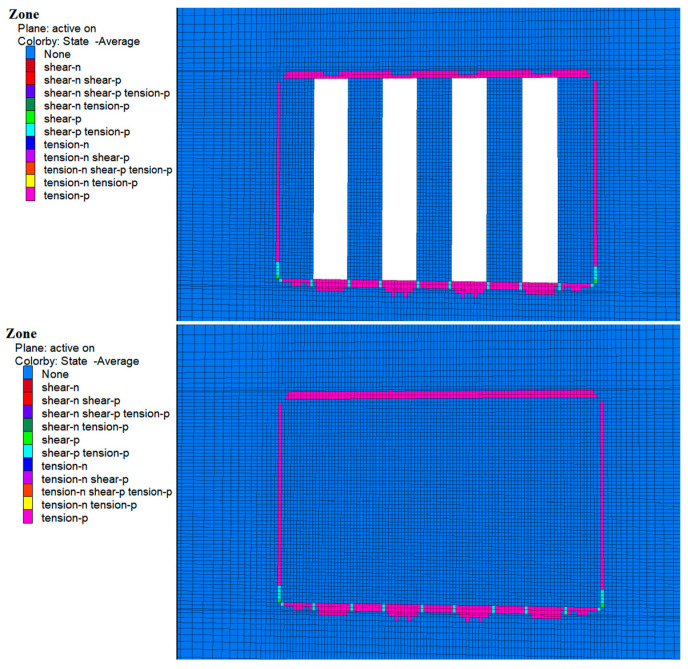
Cloud map of the 2-step mine room excavation and filling of the plastic zone in the b ore layer deposits.

**Figure 21:**
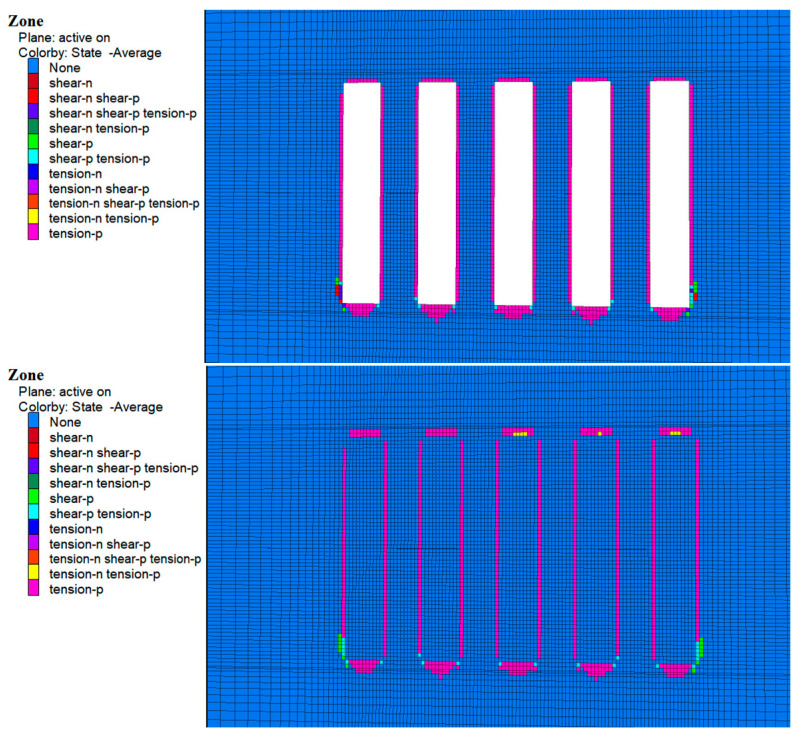
Cloud map of the one-step mine room excavation and filling of the plastic zone in the a ore layer deposits.

As shown in Figure 20 and Figure 21, following cemented fill in Room 2 of the a and b horizons, no plastic damage zones were observed in the fill material. This suggests that the cemented fill remained stable and undamaged as it withstood and transferred pressure from the surrounding rock. With continued excavation of Room 2 in the a and b horizons, the cemented fill on both sides of Room 1 was exposed and the cemented zone reappeared around the surrounding rock, mainly in the top and bottom areas. At the same time, the cemented fills on both sides showed no plastic zones, indicating that these fills remained stable and undamaged when subjected to the stresses of the overlying rock.

Following the backfilling of Room 2, a complete filling structure was formed in the mining area of the a and b ore layers. This structure played a significant load-bearing role in the overall stress structure, demonstrating that the cemented fill remained stable under these conditions.

## 5. Conclusions

The main conclusions of the research in this paper are summarized as follows:

1. After conducting a comprehensive review of current research and practical applications concerning concrete aggregate grading, it is confirmed that optimizing the grading analysis for coarse-grained mine filling aggregates can effectively be achieved through the application of the Talbol method. Subsequently, a thorough assessment of the existing grading within mining operations was undertaken, leading to the following conclusions:
  The tailing sand from the Daxin mine possesses a particle size distribution index of 0.5, meeting Fuller’s gradation requirements. However, using tailing sand alone for filling does not promote the development of cemented filler strength. Therefore, despite the satisfactory gradation index of tailing sand, the filling process necessitates its mixing with coarse aggregate to reduce cement consumption and enhance filler strength.
  The gradation index (n = 0.97) of yellow phosphorus slag exceeds Fuller’s gradation index. Clearly, the gradation of yellow phosphorus slag does not meet Fuller’s requirements. In fact, the particles of yellow phosphorus slag are too large, which can result in insufficient fine-grained content in the slurry and uneven composition. Consequently, these two filling materials must be mixed with each other to meet both coarse and fine gradation requirements effectively.
2. The results of the gradation analysis of the filling materials show the following. When mixing yellow phosphorus slag and tailing sand, their gradation conforms to the Talbol gradation equation. Specifically, for ratios of yellow phosphorus slag to tailing sand of 3:7, 4:6, and 5:5, the corresponding fit indices are 0.23, 0.26, and 0.31, respectively. Notably, the ratio of 5:5 demonstrates the closest proximity to the Fuller index. This indicates that, at this ratio, the blend of yellow phosphorus slag and tailing sand achieves the highest packing density, minimum porosity, and maximum cementation strength under equivalent conditions.
  Regarding the fitting results using the Fuller gradation formula for the mixed aggregate of yellow phosphorus slag and tailings with ratios of 3:7, 4:6, and 5:5, the coefficients of determination (R²) were 0.73, 0.80, and 0.85, respectively. To validate the reliability of these fitting results, the paper employed the mean absolute error (MAE) and mean absolute percentage error (MAPE) as metrics for precision testing. The outcomes indicated that the fitting results obtained using the Fuller gradation formula were deemed reliable.
3. According to the test results of the strength of the cementitious filler comprising yellow phosphorus slag and tailing sand, the highest strength was observed under different curing times with equal mass concentration and ash–sand ratio when the ratio of yellow phosphorus slag to tailing sand was 5:5. This was followed by the 4:5 ratio, with the lowest strength noted at the 3:7 ratio. Furthermore, this formulation also enhances the utilization of yellow phosphorus slag, thereby addressing issues related to its open storage.
4. Ensuring a minimum cemented filler strength threshold of 0.67 MPa is critical. Testing at a 71% mass concentration and 1:7 ash–sand ratio confirms that both the 5:5 and 4:6 ratios of yellow phosphorus slag to tailing sand meet this requirement. Further exploration at 72% and 73% concentrations across various proportioning configurations continues to meet strength criteria, indicating potential cost efficiencies through optimized concentration levels.
5. Filling calculations of the mine were performed by flac3d. During the excavation of the a and b layers, the stress concentration in the rock body led to plastic damage, but the cemented filling body effectively reduced the stress concentration and formed a stable stress-supporting structure. Although the filling body has limited resistance to the deformation of the top and bottom slabs, it remains stable when bearing and transferring the pressure of the surrounding rock, and no plastic damage occurs. As mining proceeds, the filling body gradually compacts and absorbs energy, providing a good stress environment for the next stage of safe mining.

## Figures and Tables

**Table 1:** Physical parameters of two kinds of filling aggregates.

Aggregate	Density (t/m^3^)	Loosen Volume Weight (t/m^3^)	Moisture Capacity (%)	Porosity (%)	Packing Compaction (%)
Tailing	2.90	1.25	13.43	44.19	55.81
Yellow phosphorus slag	2.56	1.36	3.089	45.11	54.89

**Table 2:** Verification of the accuracy of the fitting results.

	Evaluation Method	Calculated Value	Result
Comparison of grading frequency between tailings and Fuller	MAE	0.06	Reliable
	MAPE	4.6%	Reliable
Comparison of grading frequency between yellow phosphorus slag and Fuller	MAE	0.02	Reliable
	MAPE	7.1%	Reliable
The ratio of yellow phosphorus slag to tailings	Evaluation Method	Calculated Value	Result
3:7	MAE	0.065	Reliable
	MAPE	11.4%	Reliable
4:6	MAE	0.0062	Reliable
	MAPE	3.7%	Reliable
5:5	MAE	0.0042	Reliable
	MAPE	1.3%	Reliable

**Table 3:** Test scheme of filling slurry mixture ratio strength.

Aggregate Type	Aggregate Ratio	Mass Concentration (%)	Added Quantity of Concrete (Kg/m^3^)	Strength (MPa)
Yellow phosphorus slag and tailing	5:5	70, 71, 72, 73	190, 170, 150, 130	R_3,_ R_7_, R_28_
	4:6			
	3:7			

**Table 4:** Cementation strength of different ratios of cemented filling slurry at different ages.

Concentration	Ratio	Age	Fitting Result	Correlation
71%	5:5	3	$y=42.5\times 0.722\times 0.066\times e^{x1}\times {x2}^{-1.19}$	0.76
71%	5:5	7	$y=42.5\times 0.722\times 0.77\times e^{x1}\times {x2}^{-2.78}$	0.92
71%	5:5	28	$y=42.5\times 0.722\times 0.731\times e^{x1}\times {x2}^{-2.40}$	0.98
71%	4:6	3	$y=42.5\times 0.701\times 0.055\times e^{x1}\times {x2}^{-1.1}$	0.77
71%	4:6	7	$y=42.5\times 0.701\times 0.58\times e^{x1}\times {x2}^{-2.56}$	0.95
71%	4:6	28	$y=42.5\times 0.701\times 0.56\times e^{x1}\times {x2}^{-2.22}$	0.98
71%	3:7	3	$y=42.5\times 0.698\times 0.053\times e^{x1}\times {x2}^{-1.21}$	0.78
71%	3:7	7	$y=42.5\times 0.698\times 0.7\times e^{x1}\times {x2}^{-2.9}$	0.88
71%	3:7	28	$y=42.5\times 0.698\times 0.51\times e^{x1}\times {x2}^{-2.32}$	0.98

Notes: ratio: the weight ratio of yellow phosphorus slag to tailings.

**Table 5:** Filling body and rock parameters.

Designation	Density (kg/m³)	Young (MPa)	Poisson	Bulk Modulus (MPa)	Shear Modulus (MPa)	Cohesion (MPa)	Internal Friction Angle (°)	Tension (MPa)
**Hanging wall dolomite**	2680	14200	0.251	9504.69	5675.46	0.85	22.4	0.10
**Footwall dolomite**	2630	11700	0.255	7959.18	4661.35	0.92	38.4	0.12
**Ore**	2890	14300	0.253	9649.12	5706.30	0.22	45.2	0.09
**Gangue**	2660	8300	0.261	5788.01	3291.04	0.20	44.1	0.08
**Cemented fill**	1890	550	0.260	381.94	218.25	0.60	30.0	0.243

## Data Availability

The original contributions presented in the study are included in the article, further inquiries can be directed to the corresponding author.

## Appendix A. Schedules

**Table A1:** Yellow phosphorus slag + tailing sand 5:5 cemented filling foundation test strength results.

Mass Concentration (%)	Gray-To-Sand Ratio	Cement (kg/m³)	Slag (kg/m³)	Tailing (kg/m³)	R3 (MPa)	R7 (MPa)	R28 (MPa)
70	1/6	190	538.1	538.1	0.78	1.81	2.90
71	1/6	190	554.5	554.5	0.89	2.22	3.06
72	1/6	190	571.3	571.3	1.04	2.58	3.36
73	1/6	190	588.4	588.4	1.16	2.83	3.60
70	1/7	170	548.4	548.4	0.69	1.24	1.98
71	1/7	170	563.9	563.9	0.78	1.38	2.23
72	1/7	170	580.7	580.7	0.89	1.60	2.45
73	1/7	170	597.8	597.8	1.08	1.75	2.48
70	1/8	150	557.9	557.9	0.59	1.05	1.39
71	1/8	150	573.3	573.3	0.67	1.15	1.57
72	1/8	150	590.1	590.1	0.74	1.24	1.95
73	1/8	150	607.2	607.2	0.85	1.32	2.04
70	0.10	130	571.5	571.5	0.50	0.66	1.06
71	0.10	130	587.4	587.4	0.58	0.72	1.12
72	0.10	130	604.2	604.2	0.64	0.80	1.21
73	0.10	130	621.3	621.3	0.71	0.98	1.27

**Table A2:** Yellow phosphorus slag + tailing sand 4:6 cemented filling foundation test strength results.

Mass Concentration (%)	Gray-To-Sand Ratio	Cement (kg/m³)	Slag (kg/m³)	Tailing (kg/m³)	R3 (MPa)	R7 (MPa)	R28 (MPa)
70	1/6	190	432.9	649.4	0.67	1.69	2.50
71	1/6	190	446.2	669.3	0.79	1.91	2.84
72	1/6	190	459.8	689.7	0.89	2.28	2.89
73	1/6	190	473.7	710.5	0.99	2.43	3.09
70	1/7	170	442.4	663.6	0.61	1.10	1.84
71	1/7	170	455.7	683.5	0.69	1.25	2.02
72	1/7	170	469.2	703.8	0.83	1.37	2.11
73	1/7	170	483.1	724.7	0.91	1.50	2.13
70	1/8	150	450.0	675.0	0.52	0.93	1.29
71	1/8	150	463.2	694.9	0.62	1.02	1.39
72	1/8	150	476.8	715.2	0.65	1.16	1.72
73	1/8	150	490.7	736.0	0.73	1.17	1.80
70	1/10	130	459.5	689.2	0.43	0.61	0.94
71	1/10	130	472.7	709.1	0.53	0.63	1.01
72	1/10	130	486.2	729.4	0.55	0.70	1.07
73	1/10	130	500.1	750.2	0.64	0.89	1.15

**Table A3:** Yellow phosphorus slag + tailing sand 3:7 cemented filling foundation test strength results.

Mass Concentration (%)	Gray-To-Sand Ratio	Cement (kg/m³)	Slag (kg/m³)	Tailing (kg/m³)	R3 (MPa)	R7 (MPa)	R28 (MPa)
70	1/6	190	326.6	762.0	0.60	1.29	2.02
71	1/6	190	336.6	785.5	0.67	1.52	2.25
72	1/6	190	346.9	809.5	0.80	1.90	2.31
73	1/6	190	357.5	834.1	0.86	2.24	2.57
70	1/7	170	332.3	775.4	0.50	0.91	1.36
71	1/7	170	342.4	798.8	0.61	0.98	1.64
72	1/7	170	352.6	822.8	0.62	1.13	1.81
73	1/7	170	363.2	847.4	0.76	1.29	1.90
70	1/8	150	338.0	788.7	0.42	0.77	1.10
71	1/8	150	348.1	812.2	0.50	0.79	1.18
72	1/8	150	358.4	836.2	0.55	0.91	1.39
73	1/8	150	368.9	860.7	0.64	1.01	1.51
70	1/10	130	346.6	808.8	0.40	0.47	0.73
71	1/10	130	356.6	832.2	0.42	0.57	0.80
72	1/10	130	366.9	856.1	0.47	0.55	0.90
73	1/10	130	377.4	880.7	0.53	0.75	0.94

## References

[1] Li X., Du J., Gao L., He S., Gan L., Sun C., Shi Y. (2017). Immobilization of phosphogypsum for cemented paste backfill and its environmental effect. J. Clean. Prod..

[2] Li X., Zhou Z., Zhao G., Liu Z. (2008). Utilization of phosphogypsum for backfilling, way to relieve its environmental impact. Gospod. Surowcami Miner..

[3] Guo S., Zhang J., Li M., Zhou N., Song W., Wang Z., Qi S. (2021). A preliminary study of solid-waste coal gangue based biomineralization as eco-friendly underground backfill material: Material preparation and macro-micro analyses. Sci. Total Environ..

[4] Chen J.-S. (2010). Cemented backfilling performance of yellow phosphorus slag. Int. J. Miner. Metall. Mater..

[5] Cacciuttolo C., Marinovic A. (2023). Experiences of Underground Mine Backfilling Using Mine Tailings Developed in the Andean Region of Peru: A Green Mining Solution to Reduce Socio-Environmental Impacts. Sustainability.

[6] Li S., Zhao Z.M., Yu H.X., Wang X.M. (2021). The Recent Progress China Has Made in the Backfill Mining Method, Part II: The Composition and Typical Examples of Backfill Systems. Minerals.

[7] Rybak J., Adigamov A., Kongar-Syuryun C., Khayrutdinov M., Tyulyaeva Y. (2021). Renewable-Resource Technologies in Mining and Metallurgical Enterprises Providing Environmental Safety. Minerals.

[8] Huang P., Spearing S., Ju F., Jessu K.V., Wang Z.W., Ning P. (2019). Control Effects of Five Common Solid Waste Backfilling Materials on In Situ Strata of Gob. Energies.

[9] Skrzypkowski K., Gómez R., Zagórski K., Zagórska A., Gómez-Espina R. (2023). Review of Underground Mining Methods in World-Class Base Metal Deposits: Experiences from Poland and Chile. Energies.

[10] Zhou S., Li B., Zhou Y., Min C., Shi Y. (2020). Effect of phosphorus on the properties of phosphogypsum-based cemented backfill. J. Hazard. Mater..

[11] Liu X.Y., Liu X.M., Zhang Z.Q., Wei C., Zeng Q.S., Li Y.T., Ma S.L. (2023). Investigation of the Pozzolanic Activity Improvement of Yellow Phosphorus Slag with Thermal Activation. Materials.

[12] Huang T., Peng Q.K., Yu L., Li D.W. (2017). The Detoxification of Heavy Metals in the Phosphate Tailing-contaminated Soil through Sequential Microbial Pretreatment and Electrokinetic Remediation. Soil Sediment Contam. Int. J..

[13] Zhang H.L., Jin L.Z., Wu H.J., Zhang Z.Y., Yu J.X., Zhang W.J., Pan Y., Pan Z.Q. (2022). Preparation of a Novel Organic Phosphonic Acid Intercalated Phosphate Tailings Based Hydrotalcite and Its Application in Enhancing Fire Safety for Epoxy Resin. Polymers.

[14] Qian G.Q., Bai S.Y., Ju S.J., Huang T. (2013). Laboratory Evaluation on Recycling Waste Phosphorus Slag as the Mineral Filler in Hot-Mix Asphalt. J. Mater. Civ. Eng..

[15] Gnandi K., Rezaie Boroon M.H., Edorh P. (2009). The Geochemical Characterization of Mine Effluents from the Phosphorite Processing Plant of Kpémé (Southern Togo). Mine Water Environ..

[16] Su Z.N., Li X.H. (2021). Study on Preparation and Interfacial Transition Zone Microstructure of Red Mud-Yellow Phosphorus Slag-Cement Concrete. Materials.

[17] Wang L., Guo F.X., Lin Y.Q., Yang H.M., Tang S.W. (2020). Comparison between the effects of phosphorous slag and fly ash on the C-S-H structure, long-term hydration heat and volume deformation of cement-based materials. Constr. Build. Mater..

[18] He X.Y., Ye Q., Yang J., Dai F., Su Y., Wang Y.B., Bohumír S. (2018). Physico-chemical Characteristics of Wet-milled Ultrafine-granulated Phosphorus Slag as a Supplementary Cementitious Material. J. Wuhan Univ. Technol.-Mater. Sci. Ed..

[19] Hou C.H., Li L.Y., Hou L.S., Liu B.B., Gu S.Y., Yao Y., Wang H.B. (2021). Sustainable and Clean Utilization of Yellow Phosphorus Slag (YPS): Activation and Preparation of Granular Rice Fertilizer. Materials.

[20] Li Z., Wu Y., Zhang X., Li X., Zhao W., Gao W., Li H., Li Y., Yan T. (2024). Study on explosion impact pressure and damage distribution law of rock powder segmented charge. Eng. Fail. Anal..

[21] Naseroleslami R., Bakhshi J., Chari M.N., Yaghoobi M.A., Mahdi M.H. (2023). Properties of Cement Mortars Containing Phosphorous Slag. J. Mater. Civ. Eng..

[22] Uy B. (2000). Strength of concrete filled steel box columns incorporating local buckling. J. Struct. Eng..

[23] Li D.X., Shen J.L., Mao L.X., Wu X.Q. (2000). The influence of admixtures on the properties of phosphorous slag cement. Cem. Concr. Res..

[24] Li D.X., Shen J.L., Chen L., Wu X.Q. (2001). The influence of fast-setting/early-strength agent on high phosphorous slag content cement. Cem. Concr. Res..

[25] Li D.X., Chen L., Xu Z.Z., Luo Z.M. (2002). A blended cement containing blast furnace slag and phosphorous slag. J. Wuhan Univ. Technol. Mater. Sci. Ed..

[26] Gao P.W., Lu X.L., Yang C.X., Li X.Y., Shi N.N., Jin S.C. (2008). Microstructure and pore structure of concrete mixed with superfine phosphorous slag and superplasticizer. Constr. Build. Mater..

[27] Yong C.L., Mo K.H., Koting S. (2022). Phosphorus slag in supplementary cementitious and alkali activated materials: A review on activation methods. Constr. Build. Mater..

[28] Kou Y.P., Deng Y.C., Tan Y.Y., Han C.C., Song W.D. (2023). Hydration Characteristics and Early Strength Evolution of Classified Fine Tailings Cemented Backfill. Materials.

[29] Mehdizadeh H., Shao X., Mo K.H., Ling T.C. (2022). Enhancement of early age cementitious properties of yellow phosphorus slag via CO_2_ aqueous carbonation. Cem. Concr. Compos..

[30] Yin X., Ma L.P., Li K., Du W., Hou P.X., Dai Q.X., Xiong X., Xie L.G. (2023). Preparation of phosphogypsum-based cemented paste backfill and its environmental impact based on multi-source industrial solid waste. Constr. Build. Mater..

[31] Jia R.Q., Wang Q., Luo T. (2022). Understanding the workability of alkali-activated phosphorus slag pastes: Effects of alkali dose and silicate modulus on early-age hydration reactions. Cem. Concr. Compos..

[32] Mehdizadeh H., Kani E.N., Sanchez A.P., Fernandez-Jimenez A. (2018). Rheology of activated phosphorus slag with lime and alkaline salts. Cem. Concr. Res..

[33] Xie F.Z., Liu Z., Zhang D.W., Wang J.X., Wang D.M. (2020). Understanding the acting mechanism of NaOH adjusting the transformation of viscoelastic properties of alkali activated phosphorus slag. Constr. Build. Mater..

[34] Mehdizadeh H., Kani E.N. (2018). Rheology and apparent activation energy of alkali activated phosphorous slag. Constr. Build. Mater..

[35] Zhang Y.N., Zhang L., Wang Q.J., Han D., Li Z.J. (2023). Iron ore tailings, phosphate slags, and lithium slags as ternary supplementary cementitious materials for concrete: Study on compression strength and microstructure. Mater. Today Commun..

[36] Chen J.J., Guan G.X., Ng P.L., Kwan A.K.H., Chu S.H. (2021). Packing optimization of paste and aggregate phases for sustainability and performance improvement of concrete. Adv. Powder Technol..

[37] Rao B.K., Reddy M.A.K., Rao A.V. (2018). Effect of flyash as cement replacement material and pore filling material in concrete. Mater. Today Proc..

[38] Bentz D.P., Garboczi E.J., Haecker C.J., Jensen O.M. (1999). Effects of cement particle size distribution on performance properties of Portland cement-based materials. Cem. Concr. Res..

[39] Zhi X., Yang T., Zhang X., Ren Y., Deng P., Chen Y.L., Xiao Y.J. (2023). Experimental Study on the Mechanical Properties and Permeability of Cement-Stabilized Permeable Recycle Aggregate Materials. Sustainability.

[40] Yang L.X., Song X.F., Lu M.R., Xia Y.H. (2022). The mixture proportioning design of sand-containing permeable concrete based on mortar thickness of recycled coarse aggregate. Mater. Rep..

[41] Huang J., Zhang Y., Sun Y., Ren J., Zhao Z., Zhang J. (2021). Evaluation of pore size distribution and permeability reduction behavior in permeable concrete. Constr. Build. Mater..

[42] Zaetang Y., Sata V., Wongsa A., Chindaprasirt P. (2016). Properties of permeable concrete containing recycled concrete block aggregate and recycled concrete aggregate. Constr. Build. Mater..

[43] Deng H.W., Duan T., Tian G.L., Liu Y., Zhang W.Y. (2021). Research on Strength Prediction Model and Microscopic Analysis of Mechanical Characteristics of Cemented Tailings Backfill under Fractal Theory. Minerals.

[44] Lee K.J.L., Wong S.F. (2023). Multi-Objective Taguchi Optimization of Cement Concrete Incorporating Recycled Mixed Plastic Fine Aggregate Using Modified Fuller’s Equation. Buildings.

[45] Jin S., Zhang J., Han S. (2017). Fractal analysis of relation between strength and pore structure of hardened mortar. Constr. Build. Mater..

[46] Quan C.Q., Jiao C.J., Chen W.Z., Xue Z.C., Liang R., Chen X.F. (2023). The Impact of Fractal Gradation of Aggregate on the Mechanical and Durable Characteristics of Recycled Concrete. Fractal Fract..

[47] Gao S., Li Q.Y., Luo J.L. (2021). Fractal characteristic of recycled aggregate and its influence on the physical property of recycled aggregate concrete. Rev. Adv. Mater. Sci..

[48] Xu F., Wang S., Li T., Liu B., Li B.B., Zhou Y. (2020). Mechanical properties and pore structure of recycled aggregate concrete made with iron ore tailings and polypropylene fibers. J. Build. Eng..

[49] Tang W.C., Khavarian M., Yousefi A., Chan R.W.K., Cui H.Z. (2019). Influence of Surface Treatment of Recycled Aggregates on Mechanical Properties and Bond Strength of Self-Compacting Concrete. Sustainability.

